# Major sources and monthly variations in the release of land-derived marine debris from the Greater Jakarta area, Indonesia

**DOI:** 10.1038/s41598-019-55065-2

**Published:** 2019-12-10

**Authors:** Muhammad Reza Cordova, Intan Suci Nurhati

**Affiliations:** Research Center for Oceanography, Indonesian Institute of Sciences (LIPI), Jalan Pasir Putih 1, Ancol Timur, Pademangan, Jakarta Utara, Jakarta, 14430 Indonesia

**Keywords:** Environmental impact, Marine chemistry

## Abstract

As marine debris becomes increasingly prevalent and induces cascading impacts on marine ecosystems, monitoring of land-derived debris is key for identifying effective mitigation strategies. Indonesia plays a pivotal role in reducing land-derived debris into the oceans considering its extensive coastline, large population and high waste production. We present the first marine debris monitoring data from Indonesia’s capital, the Greater Jakarta area, by characterizing major sources and monthly variations of debris release at nine river outlets into Jakarta Bay between June 2015-June 2016. Our data show plastics as the most common debris entering Jakarta Bay representing 59% (abundance) or 37% (weight) of the total collected debris. Styrofoam was dominating among plastic debris, highlighting the urgency of reducing plastic and styrofoam uses. Higher debris releases during the rainy season (December-February) highlight the need to intensify river clean-up activities. We estimated an average daily debris release of 97,098 ± 28,932 items or 23 ± 7.10 tons into Jakarta Bay with considerably lower inputs from the capital compared to its neighboring municipalities. Within the plastics category, field monitoring data yield a daily plastic debris release of 8.32 ± 2.44 tons from the Greater Jakarta area, which is 8–16 times less than global-scale model estimates.

## Introduction

The presence of marine debris − a persistent, solid discarded waste in the marine environment, is pervasive in beaches, coastal waters and open oceans mainly due to land-based human activities. Marine debris pollutes the ocean from the water column to the seafloor^1–4^ with detrimental consequences for marine ecosystems^5–7^ and the economy^8,9^. Currently, there are about 7,000–250,000 tons of plastic debris resides in the world oceans^2–4^. It has been estimated that approximately 80% of marine plastic debris originates from land-based human activities^10,11^. The input of marine plastic debris from coastal areas varies substantially, depending on geographic factors related to humans (e.g., the coastal population, amount of waste generated, percentage of unmanaged waste^10^) as well as the environment (e.g., river discharge that could deliver land-derived debris into the oceans^12,13^).

Indonesia’s extensive coastline, large population and a high percentage of unmanaged waste are recipes for contributing significant amounts of land-derived debris into oceans. Studies have ranked Indonesia as the second-largest plastic waste contributor to the world’s oceans after China^10,13^. The Indonesian archipelago covers a 99,093 km-long coastline^14^. It is home to the world’s fourth-largest population (255.46 million) where a majority (57%) resides in Java Island^15^ with a concentration around the capital city of Jakarta. Indonesia produces about 200,000 tons of waste annually with only 64% reaching landfills while the rest ends up in the environment^16^. Despite the reported impacts of debris on marine organisms and fisheries in Indonesia^17,18^, long-term monitoring studies that characterize major sources and seasonal variations of debris release into marine ecosystems are lacking. Willoughby^19^ was the first to study the composition and distribution of debris in the Seribu Islands located offshore from Jakarta. Willoughby *et al*.^20^ also suggested that the island of Java may be the main source of marine debris in Indonesia. Besides being densely populated, Java Island has several rivers that serve as a conduit for land-derived debris such as plastics to reach the oceans^21^.

Under the United Nations’ Sustainable Development Goals (SDGs), the SDG 14.1 aims to “by 2025, prevent and significantly reduce marine pollution of all kinds, particularly from land-based activities, including marine debris and nutrient pollution.” In response, Indonesia has created a national action plan to combat marine plastic debris between 2017–2025 through several initiatives such as reducing land-derived plastic waste in rivers. To assess the effectiveness of ongoing initiatives in reducing marine plastic debris, it is important to conduct a spatially and temporally comprehensive marine debris monitoring in major Indonesian rivers.

**Figure 1:**
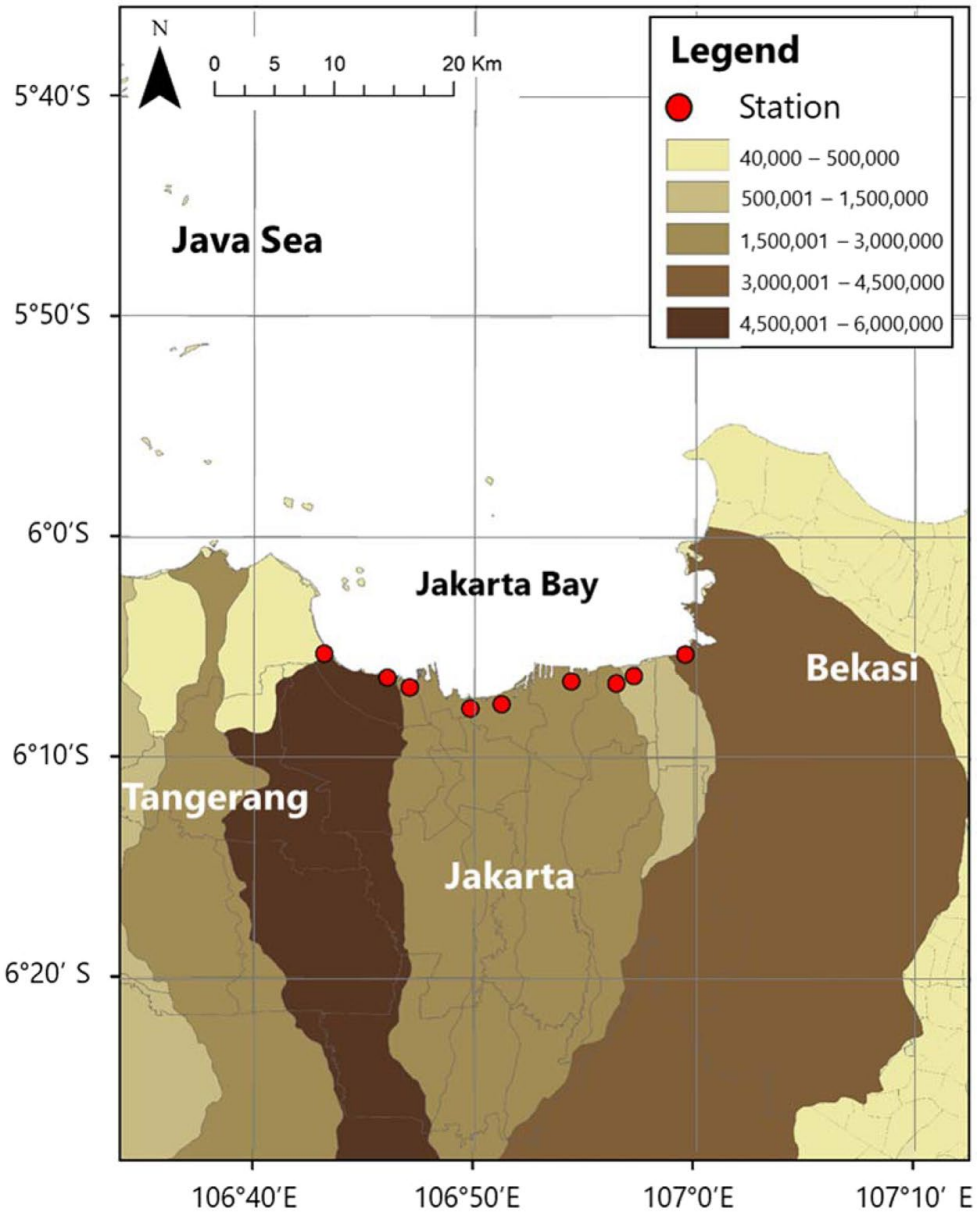
The Greater Jakarta area showing the nine river outlets (circles) into Jakarta Bay that belong to several watersheds across Tangerang, Jakarta and Bekasi. The color gradient shows the population in the watersheds.

Here, we present the first marine debris monitoring that characterized major sources and monthly variation of marine debris in nine river outlets into Jakarta Bay over the period June 2015 to June 2016 (13 months). The nine river outlets span across three municipalities in the Greater Jakarta area, which are Tangerang, Jakarta and Bekasi. The rivers belong to several watersheds with varying population pressures (Fig. 1). The Greater Jakarta area has a population of 30 million^15^ and produces solid wastes of about 6,000–7,000 tons per day^22^. With the assumption that about 10% make their ways to the oceans^23^, it is thus estimated that the amount of waste that enters Jakarta Bay may reach 600 to 700 tons daily. For our monitoring study, debris was collected, quantified by abundance into 6 types of debris (plastics, metal, glass, wood/paper, cloth/fiber, and others)^24^ and 19 categories of plastics^24,25^ (Table 1), and weighted (see Methods: Estimates of Marine and Plastic Debris Release). We compared our estimates of debris release reported by abundance (items/day) and weight (tons/day) from the three municipalities against rainfall records from nearby weather stations^26^ as well as river discharge data collected in the field (see Methods: Rainfall and River Discharge), to understand the role of hydrometeorological variability on marine debris release from the Greater Jakarta area.

**Table 1:** Percentages of debris collected at the nine river outlets into Jakarta Bay across the municipalities of Tangerang, Jakarta and Bekasi under the 19 plastic categories by abundance and weight.

Plastics categories	Tangerang		Jakarta		Bekasi	
	Abundance (%)	Weight (%)	Abundance (%)	Weight (%)	Abundance (%)	Weight (%)
Ball, Tires, Balloons, Pieces	0.03	0.12	0.09	0.44	0.02	0.08
Plastic bottles	2.96	0.93	6.96	3.28	2.71	2.39
Plastic cups	6.67	1.66	9.17	0.88	7.30	1.47
Plastic cover	2.78	0.35	4.28	0.59	4.22	0.23
Plastic match, tips, cigarettes	4.45	0.38	4.24	0.41	1.93	3.07
Thin plastic wrap	9.64	3.30	7.24	1.26	12.49	1.22
Thick plastic wrap, sack	6.39	8.82	6.41	7.32	6.79	8.17
Rubber bands, rubber pieces	1.48	0.93	5.65	3.26	1.18	0.60
Masking tape, duct tape, plastic pieces	0.19	0.04	3.02	0.48	2.90	0.27
Medicine wrap	1.67	1.56	5.60	4.48	6.10	1.91
Straw, pieces	1.85	0.40	6.23	1.54	1.98	0.23
Food boxes, plastic utensil, etc.	9.26	0.28	5.80	0.38	6.62	0.05
Shoes, sandals, gloves, cuts	11.12	*33.60	4.85	9.15	5.09	4.86
Styrofoam	*31.69	1.48	*11.47	1.48	*25.45	2.13
Rope, fishing line, fishing rod	0.93	13.94	3.81	*25.54	3.56	7.99
Plastic rope/small net pieces	4.72	2.88	4.06	7.37	6.27	1.09
Pipe, hoses, pieces	0.93	16.51	3.72	12.62	1.41	*59.55
Another plastic fault	1.85	8.51	3.02	11.51	1.37	1.46
Wrap cosmetics, toiletries, etc.	1.39	4.31	4.37	8.01	2.62	3.24

The highest percentages at each municipality are noted with asterisks.

## Results

### Major sources of marine debris into Jakarta Bay

**Figure 2:**
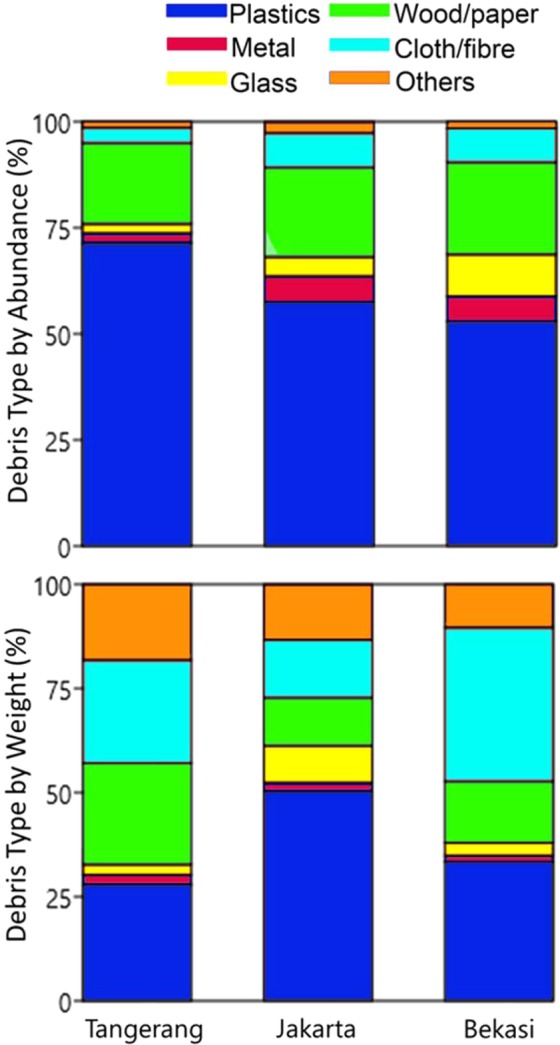
Percentages of debris type by abundance (top) and weight (bottom) in the nine river outlets into Jakarta Bay across the municipalities of Tangerang, Jakarta and Bekasi over the period June 2015 to June 2016.

Our data reveal plastics as the single most dominant debris entering Jakarta Bay (Fig. 2), which accounted for 59% by abundance (57,668 ± 16,559 items) or 37% by weight (8.32 ± 2.44 tons) of the total collected debris over the period June 2015 to June 2016. In the municipality of Tangerang, plastics represented 71% by abundance (18,273 ± 5,292 items) or 28% by weight (2.15 ± 0.88 tons). In Jakarta, plastics were 57% by abundance (19,327 ± 4,767 items) or 50% by weight (3.56 ± 0.77 tons) of the total collected debris. And in Bekasi, plastics represented 53% by abundance (20,066 ± 10,074 items) or 33% by weight (2.61 ± 1.31 tons). Debris under the wood/paper type was the second most abundant after plastics, while debris under the type of cloth/fiber was also prominent vis-à-vis weight particularly in Bekasi.

Styrofoam represented the most abundant debris within the plastics category (Table 1). By abundance, roughly 32%, 11% and 25% of the plastic debris found in Tangerang, Jakarta and Bekasi, respectively, were made of styrofoam. In the municipality of Tangerang, other abundant plastic items included shoes, sandals, gloves, cuts (11%), thin plastic wrap (10%), food boxes, plastic utensil, etc. (9%), and thick plastic wrap, sack (6%). Other abundant plastic items in Jakarta were plastic cups (9%), thin plastic wrap (7%), plastic bottles (7%) and thick plastic wrap, sack (6%). In Bekasi, thin plastic wrap (12%) and thick plastic wrap, sack (7%) were also abundant. With regards to weight, plastics belonging to the shoes, sandals, gloves, cuts category were dominant in Tangerang (34%), similarly for the rope, fishing line, fishing rod category in Jakarta (26%) and the pipe, hoses, pieces (60%) category in Bekasi.

### Estimates of marine and plastic debris release

**Figure 3:**
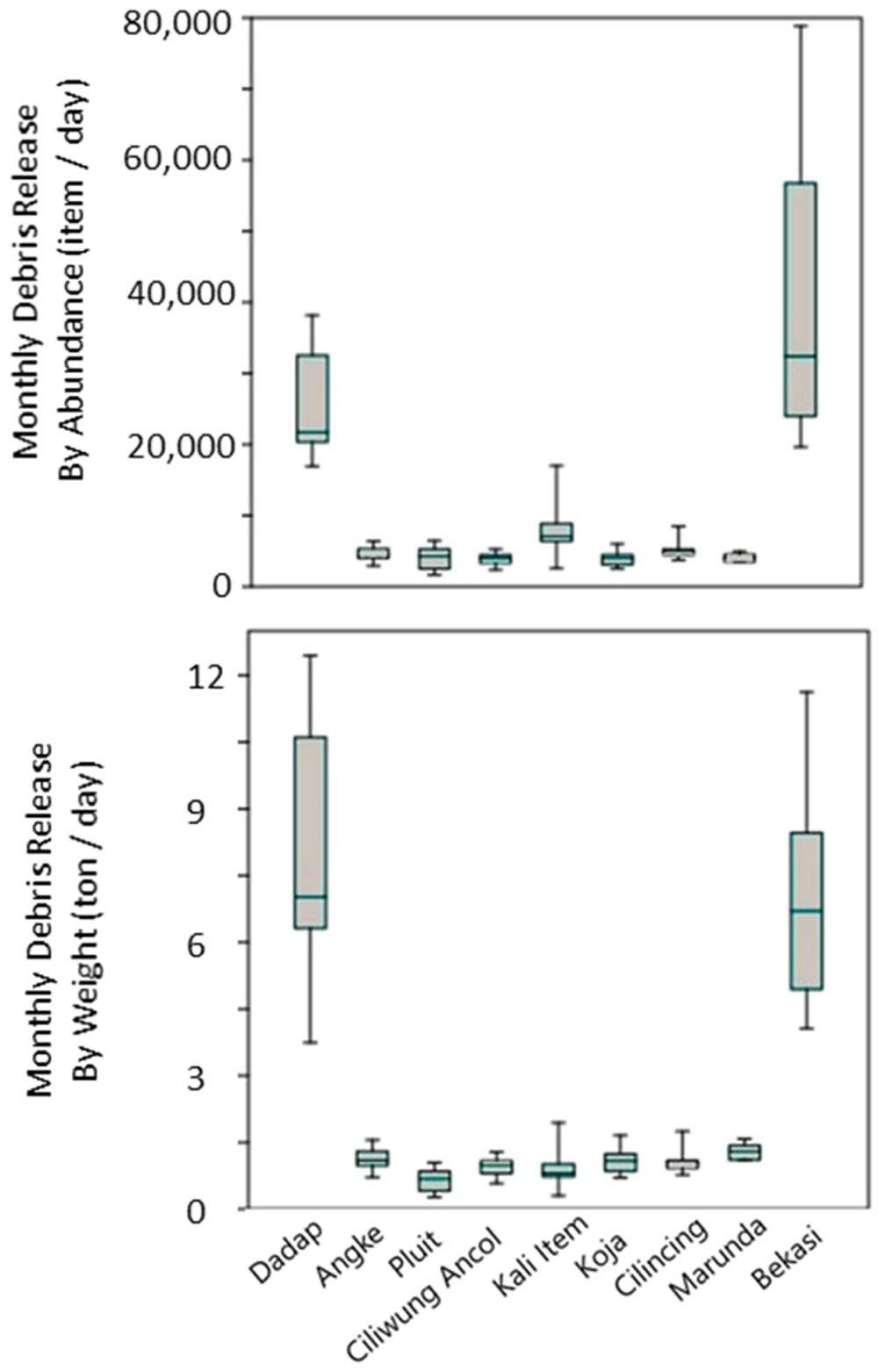
Boxplot of debris release by abundance (top) and weight (bottom) from the nine river outlets in the Greater Jakarta area into Jakarta Bay. From west to east, the river outlets are: Dadap River in Tangerang, Angke to Marunda Rivers in Jakarta, and Bekasi River in Bekasi.

We estimated daily debris release of 97,098 ± 28,932 items or 23 ± 7.10 tons of debris into Jakarta Bay via the nine river outlets with significantly lower releases from individual river outlets in the capital city of Jakarta compared to its neighboring municipalities (Fig. 3). Over the period June 2015 to June 2016, Bekasi River had the highest debris release by abundance, while Dadap River in Tangerang contributed the most in term of weight. Bekasi River delivered 37,888 ± 19,022 items or 6.67 ± 2.20 tons of debris daily, whereas Dadap River delivered 25,584 ± 7,409 items or 7.92 ± 2.71 tons of debris daily into Jakarta Bay. When combined, the seven river outlets in Jakarta had a debris release of 33,626 ± 8,375 items or 7.07 ± 1.52 tons daily into Jakarta Bay. We investigated the possibility that the lower debris releases from individual river outlets in Jakarta may relate to any variation in river discharge. We find that river outlets in the municipality of Jakarta did not have lower discharges compared to their counterparts in Tangerang and Bekasi during the study period. The river discharge data show values of 11.10 m^3^/s in Tangerang, ranging between 4.10–39.70 m^3^/s for river outlets in Jakarta, and 20.90 m^3^/s in Bekasi (see Methods: Rainfall and River Discharge). We conclude that Dadap River in Tangerang and Bekasi River indeed had higher densities of debris flowing into Jakarta Bay relative to river outlets in Jakarta.

Recognizing plastics as the dominant land-derived debris entering Jakarta Bay, we calculated daily plastic debris release of 57,668 ± 16,559 items or 8.32 ± 2.44 tons into Jakarta Bay. Bekasi River had the highest daily debris release both by abundance and weight (20,066 ± 10,074 items or 2.61 ± 1.31 tons) followed by Dadap River in Tangerang (19,327 ± 4,767 items or 2.15 ± 0.88 tons). Meanwhile, individual river outlets in the municipality of Jakarta had significantly lower debris release into Jakarta Bay that combined delivered 18,273 ± 5,292 items or 3.56 ± 0.77 tons of plastic debris daily during the study period.

### Monthly variations of marine debris releases

**Figure 4:**
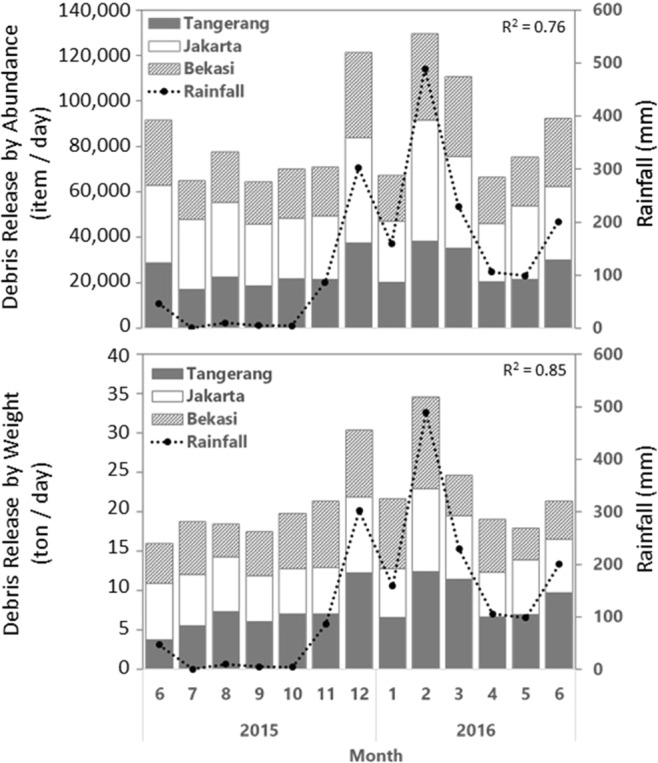
The relationships between monthly rainfall and debris release by abundance (top) and weight (bottom) from the nine river outlets in the Greater Jakarta area into Jakarta Bay. The correlation coefficients are shown.

Our year-long monitoring data revealed monthly variations in debris release into Jakarta Bay with significant correlations with rainfall amount (R^2^ = 0.76 for abundance and 0.85 for weight, N = 13, p < 0.01; Fig. 4). The highest debris release into Jakarta Bay occurred at the peak of the rainy season in February 2016 (489.25 mm of rainfall) with a total of 129,643 items or 34.56 tons of debris daily, followed by December 2015 (302.43 mm of rainfall) with a total of 121,383 items or 30.41 tons of debris daily. The lowest debris release took place on September 2015 (64,371 items daily by abundance) and June 2015 (15.98 tons daily by weight), which occurred around July 2015 that experienced the lowest rainfall amount (0.75 mm). We found no correlation between debris release and river discharge (R^2^ = 0.02, N = 9, p = 0.75 for both by abundance and weight).

## Discussion

Altogether, our monitoring data on major sources and monthly variations in the release of land-derived debris into Jakarta Bay inform stakeholders and policymakers to prioritize on particular types of debris, categories of plastics as well as months of the year to reduce land-derived debris from the Greater Jakarta area more effectively. Further, the data could help in assessing initiatives over the recent years in reducing land-derived debris through riverine channels.

Our findings showing plastics as the most dominant debris entering Jakarta Bay and styrofoam as the most abundant debris within the plastics category; convey the urgency of systematically reducing the use of plastics and styrofoam in the Greater Jakarta area. Indonesia produces 1.65 million tons of plastics yearly^27^, in which a significant portion ends up in the environment^16^. Regulations that ban the use of plastic bags in supermarkets in Tangerang, Jakarta and Bekasi have been in place since March 2019, however, single-use plastics are still used in traditional markets and online food delivery services. Styrofoam (polystyrene) is widely used for packaging foods, replacing the traditional use of organic food wraps such as banana leaves. To date, Bandung is the only Indonesian city that bans the use of styrofoam for packaging foods and beverages – an environmental initiative that the Greater Jakarta area needs to follow suit.

The significant correlation between marine debris releases and monthly rainfall variation echoes the need for more intensified river cleanup programs during the rainy season. High debris inputs entering the oceans via rivers during the rainy season have been documented in other cities worldwide^27–31^. In the case of the Greater Jakarta area, the higher debris releases may reflect not only a higher inflow of debris, but also a common practice of disposing more debris during the rainy season. Along with improved monitoring of marine debris release in major cities, our study reminds the importance of gathering environmental parameters such as rainfall, river discharge as well as water quality.

The lower debris releases from individual rivers in Jakarta compared to its neighboring municipalities during our study period cannot be explained by variations in river discharge, therefore we infer that this may reflect improved river cleanup programs in the capital city in 2015. The formation of city cleaners known as the ‘*pasukan orange*’ through Governor Regulation No. 169/2015 has been effective in cleaning up rivers in Jakarta. Albeit no available measurement of debris release before 2015, there were numerous visual documentations on markedly reduced debris in Jakarta’s rivers during this time. Long-term marine debris monitoring using relatively simple methods as we demonstrate herein could provide crucial information for assessing the effectiveness of river cleanup programs in the recent years. Similar monitoring efforts could be repeated in the area, as well as replicated in other coastal cities particularly of the developing world to provide science-based information for policymakers to combat the marine debris issue.

Our monthly monitoring of debris release into Jakarta Bay suggests a much lower value compared to existing global-scale model estimates of highly varying values. Adopting Jambeck *et al*.^10^’s assumption of ~10.1% of mismanaged waste is plastic, it can be estimated that about 55.3–73.8 tons of plastic debris enter Jakarta Bay daily. By taking into account of waste management, population density and hydrological information, Lebreton *et al*.^13^ estimated plastic inputs from five rivers into Jakarta Bay to be about ~130 tons of plastic waste per day. Whereas our *in situ* monitoring yields a considerably lower plastic debris release of 8.32 ± 2.44 tons daily that is about 8–16 times of the global-scale estimates. A simple explanation is that rivers in our study area have floating net booms in place that reduce debris releases, one of the factors that is not captured in the global-scale models. Our findings do not negate the possibility of higher debris release in the field compared to the global-scale estimates in other cities considering varying levels of local commitment to reduce land-derived debris. When combined with global models of marine debris, field monitoring at river outlets serves as ground-truth data to refine the global-scale estimates by taking into account of local solutions that are in place to reduce marine debris.

A more accurate estimate of marine debris aids the effort to meet the Sustainable Development Goal 14.1 that is to prevent and significantly reduce marine debris from land-based activities by 2025. Steps have been taken to reduce marine debris in the Indonesian waters. Under the United Nations’ SDGs, the Indonesian government has pledged to create a National Action Plan for combating marine plastic debris (Ocean Action #14387). The Coordinating Ministry of Maritime Affairs has identified 18 major cities in Indonesia including Jakarta that may contribute significantly to the marine debris problem and committed to allocate up to USD 1 billion annually to reduce 70% of plastics waste in the sea by 2025. Ultimately, public awareness instilled in the national curriculum and by the media, as well as technical solutions (e.g., waste management, recycling facilities, biodegradable plastic alternatives) are keys to accomplishing the goal.

This is the first study that characterized major sources and monthly variations of debris release at the nine river outlets in Indonesia’s capital, the Greater Jakarta area. Our work highlights the role of long-term field monitoring of marine debris in major Indonesian cities to provide crucial information for reducing land-derived debris into the oceans. Further works are needed to understand the sources, pathways and ecological impacts of marine debris using long-term field monitoring data.

## Methods

### Study location

We conducted monthly sampling of debris entering the Jakarta Bay from June 2015 to June 2016 at nine river outlets belonging to three municipalities in the Greater Jakarta area (Fig. 1). The nine river outlets are from west to east: Dadap River in Tangerang, Angke, Pluit, Ciliwung, Kali Item, Koja, Cilincing and Marunda Rivers in the capital city of Jakarta, and Bekasi River in Bekasi.

### Sampling & estimating debris release

Debris was collected from each river outlet using a 75 m-long and 1.5 m-deep net with a 5 cm mesh size. The river outlets have widths ranging between 18–64.9 m or under the length of our sampling net. The net was placed along the width of the river for 15 minutes and repeated for 3 to 6 times depending on river discharge. In our case, putting the net for more than 15 minutes at a time ran the risk of tearing the net from overfilling. The difference in sampling times between sites is accounted for in the subsequent calculation. We allocated about an hour of debris sampling at each site and sampled debris at the nine river outlets in 2–4 days.

We categorized the collected debris using a modified list of the NOAA Marine Debris Program datasheet^24^ to group the debris into six types: plastics, metal, glass, wood/paper, cloth/fiber, and others. Debris that was food waste, animal waste, too small or could not identified were put in the ‘others’ group. For the plastics group, we further classified them into 19 categories (see Table 1) as modified from existing categories^24,25^. We estimated debris release at each river outlet by abundance and weight following the formula:

$$D=\frac{N}{t}\times \frac{24\times 60\,minutes}{1\,day}$$

where D is the debris release (the number of items or weight per day); N is the number (items) or weight (ton) of the collected debris, and t is observation time at each site (minutes). The collected debris was weighed on-site using a digital scale with a 0.1 g accuracy. We removed water from the debris, therefore plastics, metal and glass debris were measured as dry weight; and semi-dry weight for wood/paper, cloth/fibre and other types of debris.

We tested the significance on the difference between monthly-averaged debris releases at the river outlets using the Kruskal-Wallis test, followed by the Mann-Whitney pairwise and Dunn’s post hoc tests using the Paleontological Statistics v.3 (PAST3) software.

### Rainfall and river discharge

Rainfall data were acquired from four nearby meteorological stations of BMKG (Tanjung Priok, Halim, Jakarta, and Cengkareng station) and a station in Bogor as an upstream region for rivers flowing into Jakarta Bay^26^.

We collected river discharge data in the field by factoring in the river area and velocity. The river area is defined by multiplying the width and depth of each river outlet. Three depth measurements were taken along the width of the river outlets. Water velocity was obtained using flow meters (Flowatch FL03 and Hydrobios 438115). River discharge measurement was carried out about 9 months out of the 13 months considering field conditions and excluding flooding events. The Dadap River has a mean discharge of 11.10 m^3^/s (minimum and maximum values of 6.05–18.22 m^3^/s), Angke is 16.10 (13.97–19.84) m^3^/s, Pluit is 11.90 (8.50–14.84) m^3^/s, Ciliwung is 39.70 (32.25–63.13) m^3^/s, Kali Item is 4.10 (2.63–6.61) m^3^/s, Koja is 17.20 (14.55–21.89) m^3^/s, Cilincing is 11.00 (6.84–13.91) m^3^/s, Marunda is 37.10 (32.94–42.83) m^3^/s, and Bekasi is 20.90 (19.08–25.67) m^3^/s.

Correlations between monthly debris release with rainfall variability and river discharge are calculated using Microsoft Excel and PAST3 software.
